# Bifunctional metavanadate promoted chitosan/cassava biopolymer films with photo-switchable wetting properties: unveiling the surface restructuring mechanism†

**DOI:** 10.1039/d4ra08196j

**Published:** 2025-03-11

**Authors:** Pongpop Sangsawang, Chayada Wisuttirattanamanee, Nichaphat Aueng-Aree, Anawat Thivasasith, Somlak Ittisanronnachai, Chokchai Kaiyasuan, Pawarisa Ngamroj, Natthakit Phophuttharaksa, Pattarapon Tanalikhit, Natputthiya Chavanalikigorn, Yutichai Mueanngern

**Affiliations:** a Department of Chemistry, Kamnoetvidya Science Academy 999 Moo 1, Pa Yup Nai, Wang Chan Rayong 21210 Thailand natputthiya.c@kvis.ac.th yutichai.m@kvis.ac.th; b Frontier Research Center (FRC), Vidyasirimedhi Institute of Science and Technology 555 Moo 1, Pa Yup Nai, Wang Chan Rayong 21210 Thailand; c School of Molecular Science and Engineering (MSE), Vidyasirimedhi Institute of Science and Technology 555 Moo 1, Pa Yup Nai, Wang Chan Rayong 21210 Thailand; d Department of Physics, Korea Advanced Institute of Science and Technology Daejeon 34141 Republic of Korea tanalikhit.p@kaist.ac.kr

## Abstract

Biopolymer films derived from starch and chitosan were soaked in vanadium salt solutions to produce vanadium metallopolymer films. Visible light irradiation induces significant color shifts from yellow to green due to changes in the oxidation state of vanadium. The material was observed to undergo dramatic structural changes upon incorporation of vanadium, with further restructuring occurring after visible light illumination. Metallopolymer films exhibited enhanced hydrophobic properties, which were further amplified when the material was irradiated with visible light, resulting in water contact angles up to 103°. X-ray photoelectron spectroscopy (XPS) measurements reveal that photoirradiation reduces vanadium metal from the 5+ (VO_3_^−^) oxidation state to lower oxidation states. Initially, V^5+^ (VO_3_^−^) interacts electrostatically with –NH_3_^+^ moieties in chitosan. These interactions were diminished following photoreduction as the formation of reduced species such as V^4+^ (VO^2+^) decreases the interaction of vanadium (previously V^5+^) with –NH_3_^+^. As the biopolymer chain breaks free from vanadium, interactions between neighboring polymer strands increase, leading to significant shifts in biopolymer surface structuring. Atomic force microscopy (AFM) measurements showed high root mean square (RMS) roughness values in starch-chitosan control films due to free interactions between biopolymer chains. Upon vanadium soaking, the chains were pulled inward by electrostatic attraction, which created a constraint that reduced the configurational states of the polymer and prevented the chains from interacting with neighboring polymer chains, significantly lowering RMS roughness. After photoirradiation, the electrostatic forces became repulsive, which released the polymer from this constraint and led to a slight increase in RMS roughness. The newly structured surface, dominated by high-frequency features, aligns well with the hydrophobicity model being developed in this work. To verify the reversible nature of the film's surface properties, irradiation and oxidative treatment cycles were conducted, and the contact angle of water was shown to drastically cycle from >100° following irradiation to ≈60° after oxidative treatments. This reversible property provides prospects and design parameters for the fabrication of future smart photo-switchable biopolymer films.

## Abstract 2

Reversible photoreduction of vanadium in chitosan-starch film yields a hydrophobic surface due to photoinduced restructuring templated by biopolymer-vanadium electrostatic forces.

## Graphical abstract

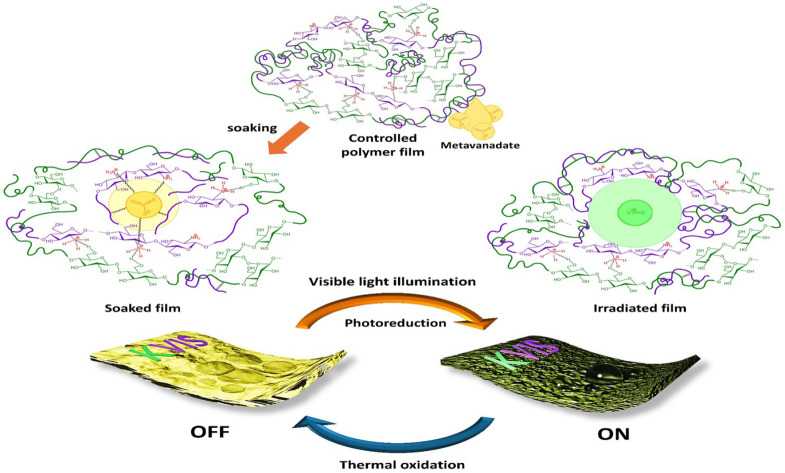

## 1. Introduction

Biomass waste accounts for over 100 billion metric tons of global waste generated each year.^1,2^ Conversion of leftover biomass to higher-value products has been at the center of several studies in the past decades.^3–5^ Commercial polymers derived from petroleum feedstocks exhibit strong physicochemical properties with high mechanical and thermal stability.^6–8^ Despite these advantages, petroleum-based products are impervious to microbial enzymes involved in degradation. This leads to significant environmental issues as these materials persist in the environment for extended periods.^9–11^ Biopolymers derived from polysaccharides provide an alternative solution to polyolefin films and have been extensively studied due to their biodegradability.^12–14^ However, these biopolymers suffer from poor barrier properties, limited mechanical and thermal stability, and high susceptibility to dissolution.^15–17^ Starch, a linear amylose structure and a branched amylopectin structure, can be easily modified with other polysaccharide chains to enhance film durability.^18,19^ Deacetylation of chitin leads to the formation of β-linked d-glucosamine and *N*-acetyl-d-glucosamine molecules, known as chitosan.^20–22^ Chitosan is commonly used to modify starch's structural properties *via* cross-linking hydroxyl groups from starch with amine groups from chitosan *via* the formation of hydrogen bonds to yield new polymer blends with strengthened structural properties.^23–28^

Biopolymers, when infused with metal ions to enhance their physical properties, transform into what is termed metallopolymers^29,30^ Harris *et al.* studied the interaction of Cu metal in electroplastic hydrogels. Here, Cu metal electrochemically modulates biopolymer films, transitioning between a soft state (Cu^+^) and a hard state (Cu^2+^).^31^ Haddad *et al.* demonstrated a significant increase in the tensile strength of pectin-chitosan films upon vanadium introduction. A photoresponse was reported as the film changed from yellow to blue upon visible light irradiation, which indicated a change in vanadium oxidation state.^32^ As with d-block metals, vanadium's electron configuration of [Ar]4s^2^3d^3^ allows for multiple oxidation states and metal–ligand interactions.^33,34^ Metal additives can promote interactions between polymer chains, leading to structural changes within the material. Due to its structurally switchable property, this study focuses on bifunctional surfaces, where hydrophobic and hydrophilic states can modulate metal–biopolymer interactions using low-energy stimuli.^35,36^ Lim *et al.* fabricated roselike V_2_O_5_ nanostructures, which were shown to exhibit superhydrophilic properties upon ultraviolet irradiation while reverting to a superhydrophobic surface under dark conditions.^37^ The fabrication of analogous soft materials with similar bifunctional properties is potentially useful in flexible coatings for commercial purposes.^38,39^

Here, biopolymer films composed of starch from cassava and chitosan were fabricated using previously reported protocols with modifications.^32,40^ Starch's flexibility along with chitosan's strength-enhancing properties facilitate the formation of robust biopolymer films.^40^ The films were then soaked in a solution of sodium metavanadate to introduce vanadium into the material matrix. Metavanadate (VO_3_^−^) is known to initially interact with –NH_3_^+^ groups in chitosan (formed following acid protonation) *via* electrostatic interactions.^41^ Upon soaking in vanadium, cassava-chitosan films (CC films) immediately turned yellow and formed vanadium-soaked cassava-chitosan films (VCC films). VCC films were then irradiated with white light, leading to the formation of green-colored films. This color change indicates a change in the oxidation state of vanadium as new interactions between vanadium and biopolymer moieties are formed.^32,41^ SEM images reveal dramatic shifts in the surface structure as the film coalesces to form an uneven morphology after the introduction of vanadium. The material then undergoes further structural changes following visible light irradiation. As the surface structure of the film is drastically altered, contact angle measurements were conducted to determine changes in film surface properties. Contact angle measurements reveal a strong photoresponsive behavior, with vanadium-incorporated films becoming significantly more hydrophobic in comparison to control films. Surface hydrophobicity was further enhanced in the case of irradiated films, where angles of >100° were observed for vanadium-soaked irradiated films. X-ray photoelectron spectroscopy (XPS) analysis indicates a more reduced vanadium surface. Here, the concentration of V^5+^ (VO_3_^−^) decreased, followed by an increase in the concentration of more reduced vanadium species. For example, V^4+^ or oxovanadium (VO^2+^) contains a 2+ charge, which, in contrast to V^5+^, interacts with –NH_3_^+^*via* repulsive forces. As vanadium is photoreduced, attractive interactions of VO_3_^−^ with –NH_3_^+^ moieties from the biopolymer are diminished due to repulsive forces between reduced vanadium species and NH_3_^+^ groups in chitosan. As the biopolymer detaches from vanadium, the interaction between the polymer chains becomes more dominant as the polymer is no longer constrained by electrostatic interactions with vanadium. This leads to increased polymer entanglement and a change in surface morphology.^42,43^ Atomic force microscopy (AFM) confirms this change, as restructuring at the surface templates the formation of pin-like structures, which have been reported to lead to surfaces with increased hydrophobic properties. Such surfaces adhere to the Cassie–Baxter equation, where high-frequency structural domains along the surface trap air pockets.^44–46^ Upon the formation of a liquid–solid interface with the surface, the contact of the water droplet is minimized, leading to lower wettability.

To demonstrate the material's bifunctionality, the material was cycled between a hydrophilic surface state using oxidative treatments and a hydrophobic state using photoreduction. The material oscillated between these two surface states over several cycles. This property provides the prospect of a multifunctional, low-cost, biodegradable material, which can be tailored to specific applications *via* low-energy stimuli.

## 2. Materials and methods

### 2.1. Materials

Chitosan (50 000–190 000 Da) was purchased from Sigma-Aldrich. All starch precursors were food-grade cassava starch.

### 2.2. Synthesis of chitosan-starch films

The synthesis followed previously reported protocols with some modifications.^32,40^ 0.6 g of chitosan was dissolved in 25 mL of 1% (w/v) hydrochloric acid solution. 0.2 mL of glycerol was then added to the previous solution. 1.2 g of cassava starch was dissolved in 30 mL of deionized water and heated at 80 °C under continuous stirring at 300 rpm until gelatinization. After 15 minutes of heating, the cassava starch solution was combined with the chitosan solution at 80 °C, and the mixture was stirred at 600–800 rpm for 10 minutes. The solution was filtered through a double-layer cheesecloth and poured onto a 400 cm^2^ glass plate. The samples were left to dry for 24 hours before collection.

### 2.3. Preparation of vanadium-soaked and irradiated vanadium films

0.243 g of sodium metavanadate (NaVO_3_, Sigma-Aldrich) was dissolved in 100 mL of deionized water at 60 °C. The chitosan-starch film was soaked in the solution for 5 minutes, then air-dried under dark conditions at room temperature for 24 hours. To yield irradiated vanadium films, vanadium-soaked samples were placed at 5 cm from a commercial 1000 W white LED panel for 20 hours to ensure maximum exposure to irradiation.

### 2.4. Preparation of irradiated vanadium films for reversibility studies

Irradiated vanadium films were divided into smaller pieces for reversibility studies. All films underwent thermal oxidation at 60 °C for 8 hours in a heating oven. Following thermal oxidation, one sample was removed for contact angle measurements. The remaining films were again exposed to visible light for 8 hours, after which another sample was removed for contact angle measurements. This process was repeated for 3.5 cycles.

### 2.5. Characterization

Film samples were cut into 1 cm^2^ pieces and analyzed using a PerkinElmer Spectrum-Two FT-IR spectrometer to investigate the interactions between vanadium and the biopolymer. Scans were collected over the range of 450 to 4000 cm^−1^.

Scanning Electron Microscopy (SEM) was conducted using a JEOL JSM7001F microscope. Samples were cut into 1 cm^2^ pieces and sputter coated with a Pt source for 3.5 minutes to minimize charging effects. Samples were loaded onto a vertical sample holder for cross-sectional analysis. Elemental analysis was carried out using an Oxford Instrument X-ray fluorescence spectrometer (EDAX X-Max) to quantify the presence of vanadium in the films.

Atomic force microscopy images were collected on an AFM Park NX10 system in non-contact mode to avoid sample damage.

JEOL/JPS-9010 MC X-ray photoelectron spectroscopy system (XPS) equipped with a Mg Kα X-ray source (1253.6 eV) was used to analyze the film samples. Data collection was done at an accelerating voltage of 15 kV with a power output of 25 W. The instrument's base pressure was 10^−10^ mbar.

Contact angle measurements were performed using a contact angle goniometer from Ossila Instruments. A water droplet was placed on the surface of the films at controlled volumes using a 100 μL micropipette, and the contact angles were accurately measured using ImageJ software. All measurements were done in triplicate. For reversibility measurements, the VCC-IR film was placed in a drying oven at 60 °C for 8 hours, followed by exposure to visible light for another 8 hours, with contact angles measured after each step. This process was repeated for 3.5 cycles to track reversibility.

## 3. Results and discussion

### 3.1. FT-IR and SEM characterization of vanadium-soaked starch-chitosan film samples

**Fig. 1:**
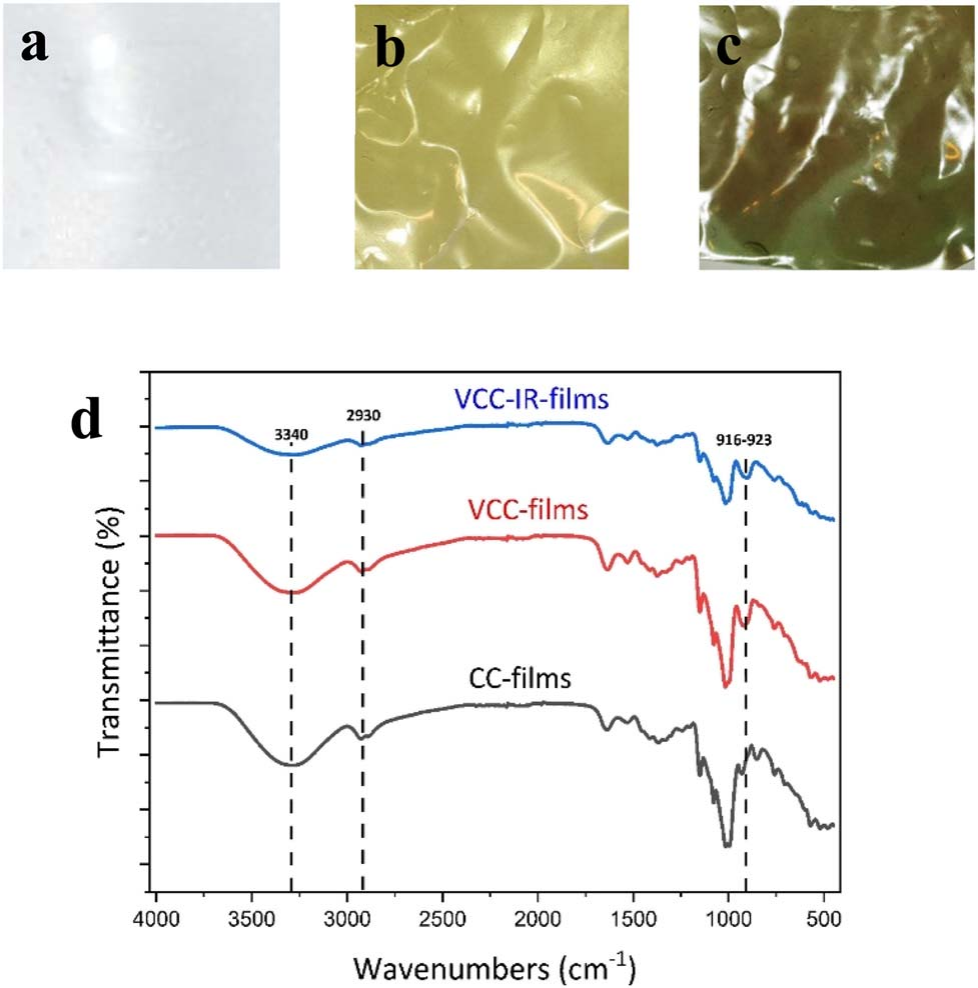
(a–c) Show images of CC, VCC, and VCC-IR film samples. (d) Shows FT-IR spectra for CC films, VCC films, and VCC-IR films. Vibrational stretches correspond to O–H and N–H stretching at 3340 cm^−1^, C–H stretching at 2930 cm^−1^, and V–O vibrations at 916–923 cm^−1^.

To study the chemical and structural transformations, three samples were fabricated: bare cassava starch-chitosan control films (CC films), vanadium-soaked films (VCC films), and vanadium-soaked films irradiated under visible light (VCC-IR films). Upon soaking in vanadium, VCC films turn yellow, which is indicative of successful incorporation of vanadium into the biopolymer. Subsequent visible light irradiation renders the films green, suggesting a change in the valence state of vanadium species as new interactions with the biopolymer form.^32,47–50^ Film colorations are shown in Fig. 1a–c, corresponding to CC, VCC, and VCC-IR films, respectively. Fig. 1d shows FT-IR analysis of CC, VCC, and VCC-IR films. CC films exhibit characteristic peaks for starch and chitosan, including O–H and N–H stretching at 3340 cm^−1^, and C–H stretching at 2930 cm^−1^. Furthermore, a strong V–O feature at 916–923 cm^−1^ is present in both VCC and VCC-IR film samples, indicating successful incorporation of vanadium into the biopolymer.^51,52^

**Fig. 2:**
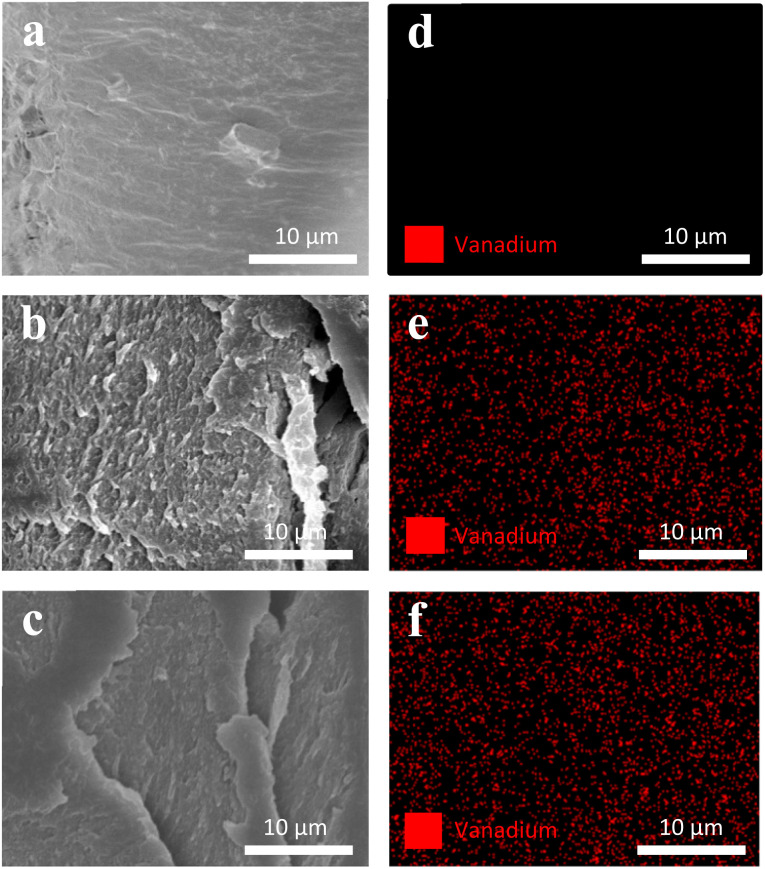
(a–c) Show SEM micrographs of CC, VCC, and VCC-IR films, respectively. Corresponding (d–f) elemental analysis (EDAX) of CC, VCC, and VCC-IR films show even distribution throughout VCC and VCC-IR film samples.

Fig. 2 show SEM-EDAX analysis of the three film types. CC films showed no restructuring at the surface (Fig. 2a). In contrast, VCC films showed increased surface heterogeneity, with the polymer matrix coalescing into larger structural domains (Fig. 2b). This type of restructuring is consistent with other studies where metal ions were incorporated into biopolymers.^53,54^ Upon irradiation, the surface morphology of the films changed further, as seen in Fig. 2c, suggesting changes in vanadium–polymer interaction.

EDAX analysis was utilized to confirm the distribution of vanadium within the polymer matrix. Fig. 2d–f show EDAX analysis of vanadium for CC, VCC, and VCC-IR films, respectively. Vanadium is shown to be uniformly distributed throughout both VCC and VCC-IR film samples.

### 3.2. Contact angle measurement experiments

**Fig. 3:**
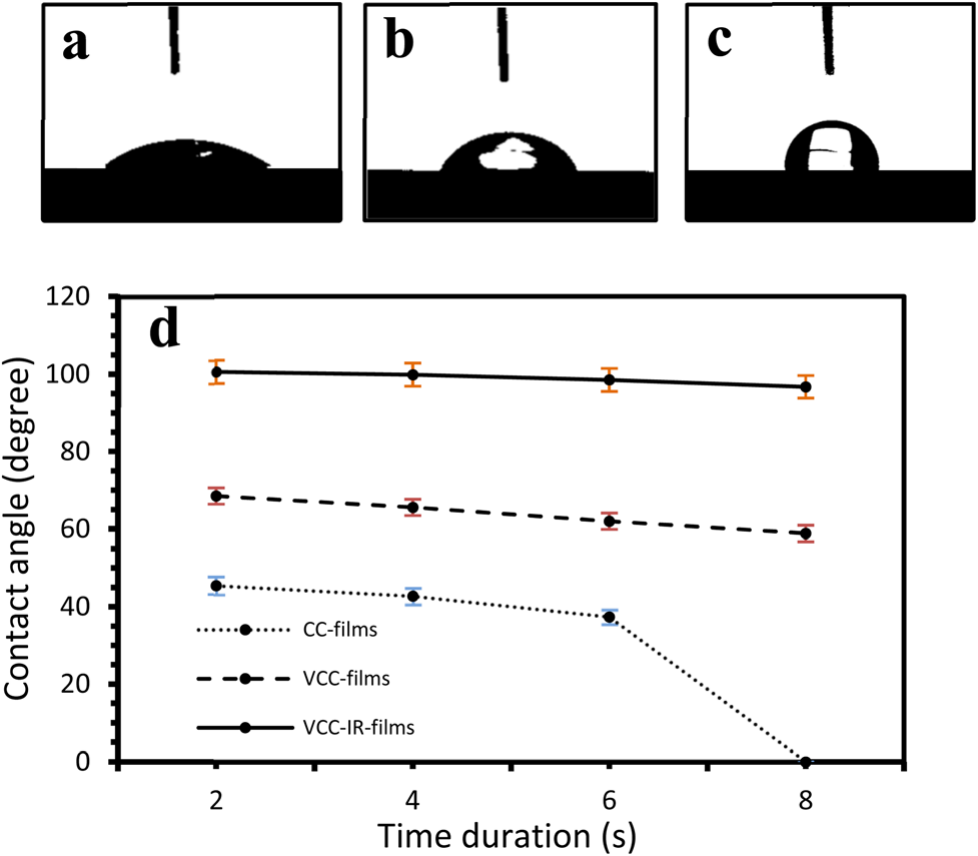
Show surface wettability properties of (a) CC films, (b) VCC films, and (c) VCC-IR films. Here, the contact angle increases from 35° to 60° to 103°, respectively. (d) Show contact angle measurements at various time points from 0 to 10 seconds.

Contact angles of water droplets were measured using a contact angle goniometer on CC, VCC, and VCC-IR films, as shown in Fig. 3. Fig. 3a–c show the contact of a single water droplet on CC, VCC, and VCC-IR films. Fig. 3d shows time profiles of surface wettability for the three samples. High wettability and low contact angles in CC film samples were expected due to the interaction between hydroxyl groups in starch/chitosan and water, leading to high wettability. It is observed that the water contact angle of CC film drastically decreases to 0° after 6 seconds. Previous studies, by Haddad *et al.*, have shown that chitosan-pectin films in the absence of vanadium readily disintegrate in water, whereas vanadium-soaked films were significantly more stable. Thus, CC films are expected to show lower water barrier properties, as the water droplet is readily incorporated into the polymer, leading to a dramatic reduction in the observed contact angle, as seen in Fig. 3d. Modifying starch with vanadium increases the contact angle by approximately 70%, from 35° to 60° as seen in Fig. 3d. Hydrophobicity arises from contact angle hysteresis between the water droplet and the underlying surface. This may be due to chemical modifications at the surface or changes in surface structuring.^44,55^ Surface restructuring can induce air-filled cavities between heterogeneous domains of microstructures along a surface, forming a solid–gas–liquid interface known as air pockets. Such microstructures have been shown to minimize contact area with water droplets, resulting in decreased surface wettability.^44–46^ The increase in contact angle due to a change in surface structure aligns with SEM micrographs in Fig. 2, which show significant surface restructuring from CC to VCC films (Fig. 2a and b). Contact angle increased to 103° following irradiation of VCC films to form VCC-IR films. This increase corresponds to additional morphological changes, as observed in SEM analysis (Fig. 2c). As surface restructuring can also lead to a change in material surface area, gas adsorption abilities for each sample were assessed. Fig. S1† shows gas uptake experiments of the biofilms, demonstrating increased adsorption of gas-phase alcohol molecules in the order CC films < VCC films < VCC-IR films. Increased gas-adsorption indicates a change in the accessible surface area of the material due to drastic shifts in surface morphologies.

### 3.3. XPS measurement

**Fig. 4:**
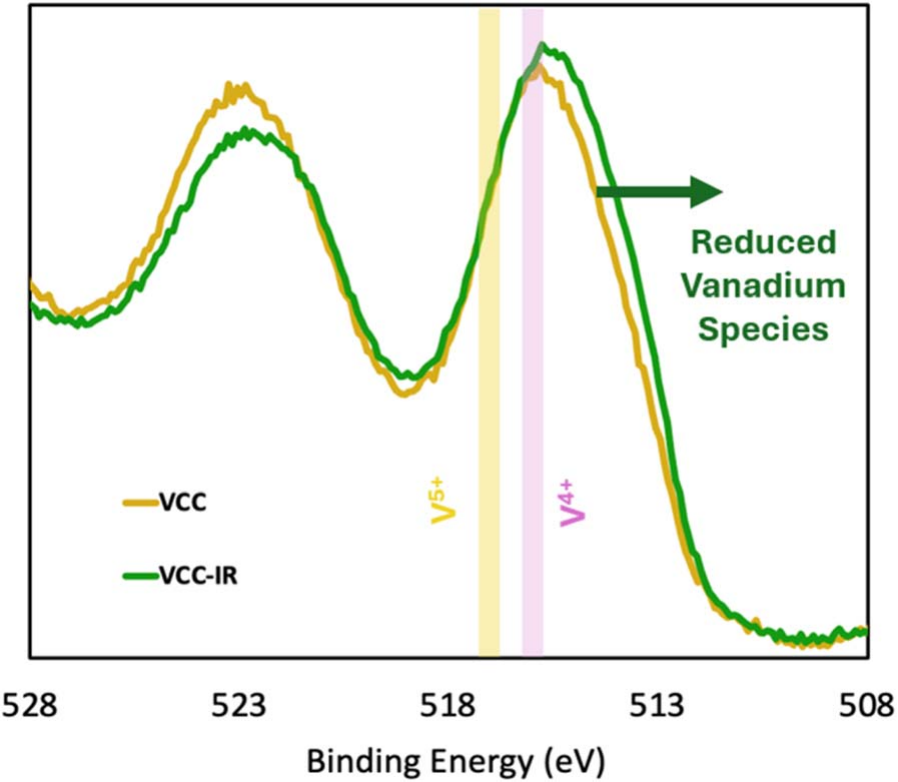
Shows V 2p spectra for VCC and VCC-IR film samples. Vanadium is observed to be more reduced in the case of VCC-IR. A mild 4+ shoulder highlighted in yellow is observed in the case of VCC-IR along with a shoulder feature at lower binding energies, belonging to reduced vanadium species: V^3+^ and V^2+^.

To investigate changes in the valence state of vanadium before and after irradiation, XPS spectra were collected for both irradiated (VCC-IR films) and non-irradiated (VCC films) biopolymer films. Fig. 4 shows XPS spectra for VCC films and VCC-IR films. Peaks were not deconvoluted due to O 1s baseline contributions to V 2p_½_, preventing proper constraints of vanadium peaks to spin–orbit area ratios. To interpret the data, noticeable features in the V 2p spectra were instead pointed out, as shown in Fig. 4. Here, a shift to lower binding energies of the V 2p spectrum post irradiation indicates the formation of more reduced vanadium species.^56^ Reduction of VO_3_^−^ (V^5+^) to vanadium species such as VO^2+^ (V^4+^) results in vanadium species which are unable to sustain electrostatic interactions with amino moieties of chitosan. Reduced vanadium species can be observed as a strong shoulder feature present in VCC-IR, as seen in Fig. 4. These contributions can result from the presence of V^3+^ and V^2+^, these species can include neutral vanadium oxide species such as V_2_O_3_ (V^3+^) and VO (V^2+^).^57^ However, a decrease in vanadium's valence state, while leading to weaker electrostatic interactions, can free up polymer chains from vanadium, increasing polymer–polymer interactions. Interactions between vanadium and the polymer matrix play a critical role in shaping the surface morphology of the material.

### 3.4. Atomic force microscopy measurements

**Fig. 5:**
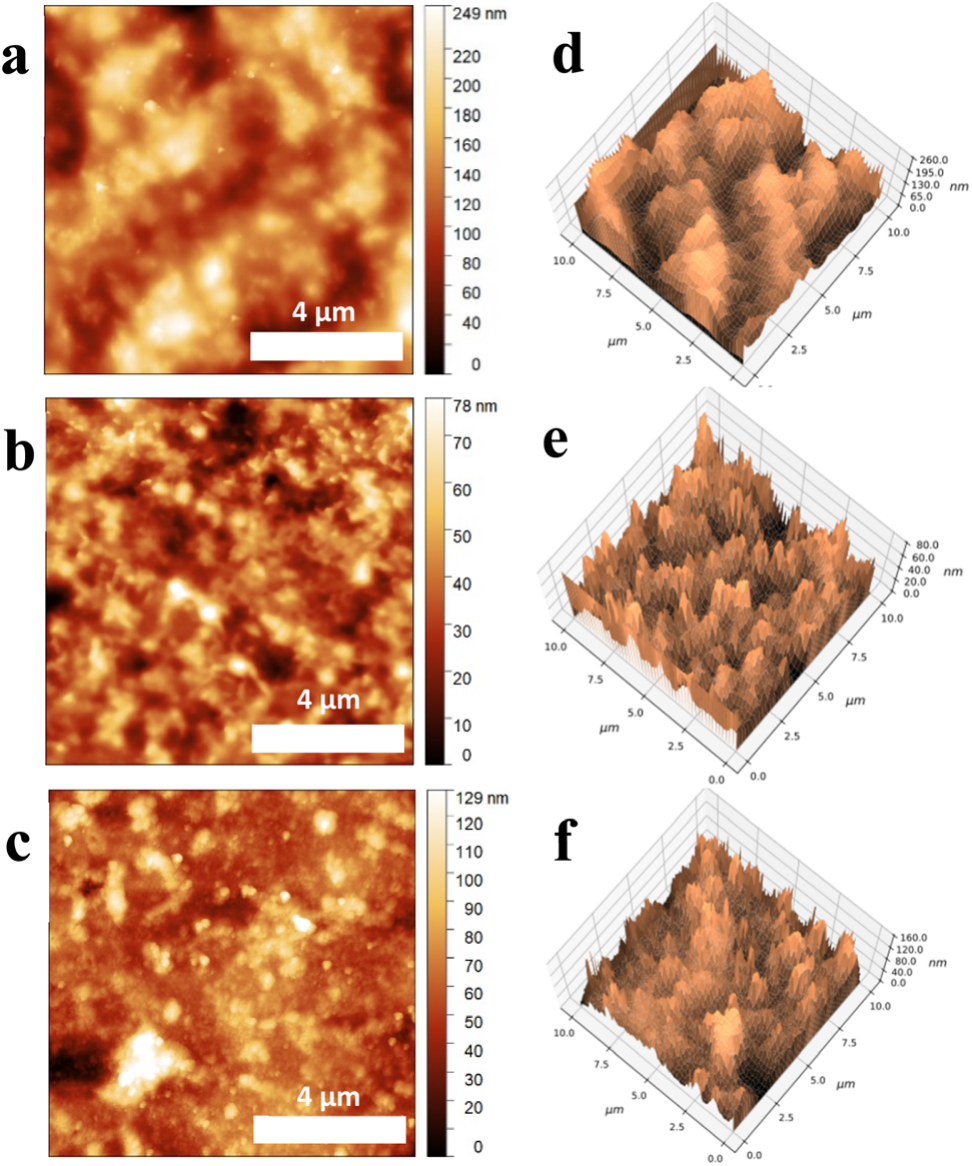
AFM micrographs of (a) CC films, (b) VCC films, and (c) VCC-IR films, with corresponding 3D AFM images for (d) CC films, (e) VCC films, and (f) VCC-IR films. Corresponding RMS roughness values are 40 nm, 12 nm, and 18 nm, as shown in (a–c) for CC, VCC, and VCC-IR films, respectively.

Surface topography and roughness of the biopolymer films were characterized using AFM measurements. Fig. 5a–c show AFM images of CC, VCC, and VCC-IR films, indicating topographical differences between the samples.

The corresponding 3D AFM reconstructions, shown in Fig. 5d–f, provide a detailed view of surface morphology; they highlight differences in the film structure. The root mean square (RMS) roughness, referring to the effective height differences between peaks and voids on the film's surface structure, for the films is 41, 12, and 18 nm for CC, VCC, and VCC-IR films, respectively. AFM analysis shows that upon incorporation of vanadium into the polymer matrix, the RMS roughness decreases from 41 nm (CC) to 12 nm (VCC). This fourfold reduction in RMS roughness indicates that the macromolecule chains are highly sensitive to vanadium interactions. As vanadium is introduced and electrostatic interactions are formed, the polymer chains interact with vanadium which is dispersed throughout the material. Electrostatic attraction “locks” the polymer into place with vanadium, limiting the degrees of freedom for which the polymer chains can interact with each other. This results in lower height dimensions and subsequently a reduction in the RMS value from 41 nm to 12 nm. As photoreduction occurs, the RMS roughness increases to 18 nm for VCC-IR films. This is reasonable, as an increase in RMS roughness implies that the polymer chains no longer interact with vanadium and are free to entangle with neighboring polymer strands. It can thus be said that irradiation of the film works to diminish the electrostatic interaction between the vanadium species and chitosan, leading to increased interactions between the polymer chains themselves. This minimization is reflected in both a change in surface structure and an increase in RMS roughness closer to that of pure CC films for VCC-IR films. The high RMS roughness in CC films indicates strong interactions between biopolymer chains, resulting in large merging domains of starch and chitosan at the surface. Upon vanadium incorporation (Fig. 5e), these structures transform into sharper, lower-height features. Following photoreduction, the surface morphology further evolves into high-frequency, pin-like structures, as seen in VCC-IR films (Fig. 5f). The morphology in Fig. 5f aligns with the Cassie–Baxter equation for hydrophobic surfaces, where air gaps between surface features reduce wettability by minimizing water–substrate interaction.^44,46^

### 3.5. Proposed mechanism

**Fig. 6:**
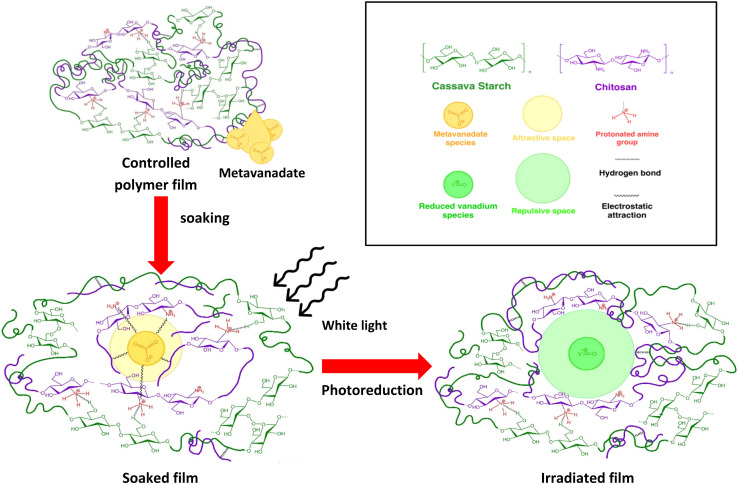
Mechanism of vanadium interaction with biopolymer films: metavanadate species (yellow circles) are introduced into cassava starch-chitosan polymer films (CC films) *via* soaking. Upon white light irradiation, photoreduction occurs, reducing the vanadium species (which become green circle) and transforming the soaked film into the irradiated film. The effective volumes (areas in 2D) of vanadium interaction in both stages, which vary as the interaction type changes from attractive to repulsive, are represented by lighter-colored circles. The inset at the top right explains the symbols.

In Fig. 6, the biopolymer shows an extremely high degree of entanglement due to numerous hydrogen bonds between –OH groups in starch and –NH_3_^+^ groups in chitosan, as well as from the entropic influence. These hydrogen bonds can occur randomly in the controlled polymer films (CC films). Introducing vanadium, by soaking, leads to metavanadate ions (VO_3_^−^), represented by the yellow circle, binding to the polymer matrix *via* electrostatic interactions (curvy lines inside the light-yellow circle) with protonated amine groups in the soaked film (VCC films). The polymer displays a low degree of entanglement because it is constrained by the attractive potential. After photoreduction, metavanadate can no longer maintain its net negative charge. This shifts the potential between vanadium and the biopolymer from an initially attractive potential (light-yellow circle) to a repulsive potential (same charge as the polymers), represented by the light-green circle. This results in higher entanglement of biopolymer chains as the unconstrained polymers are free to explore the configurational states as in CC films. This change in polymer entanglement leads to the restructuring of the entire polymer matrix in the irradiated film (VCC-IR films).

It can be seen in Fig. 6 that the attractive space (light-yellow circle) is smaller than the repulsive space (light-green circle). The underlying reason is that, in the case of the attractive space, all forces involved are of the attractive nature, and only the excluded volume acts as a repulsive force against shrinkage. As the polymers move closer to the electrostatic source, the attractive force strengthens. We define the effective radius at the point where the excluded volume balances the electrostatic attraction at small distances. However, in the repulsive case, there are both excluded volume repulsion and electrostatic forces, which become very strong near the potential source, causing the repulsive space to occupy more volume.

The initial attractive electrostatic interactions and hydrogen bonds create local entanglements, which pull the chains toward the center of the electrostatic potential, restricting the polymer chains from freely exploring random arrangements. After reduction, the repulsive forces spread the chains, which increases entanglement and enhances hydrophobicity. This is reflected in the RMS roughness changes, which align with the Cassie–Baxter equation. However, the statistical model proposed in this work, based on the aforementioned mechanism, further explains these RMS results and other outcomes beyond what is accounted for by the Cassie–Baxter equation. This model will be mathematically explored in the following analysis.

To explain this phenomenon, we propose a model that describes the system's behavior using statistical mechanics. We first consider entropy to explain the film's surface morphology at each stage and then define the partition function to explain the film's hydrophobic properties.

For entropy, we treat the CC films as an ideal system where the polymers follow random walk alignments, giving the entropy
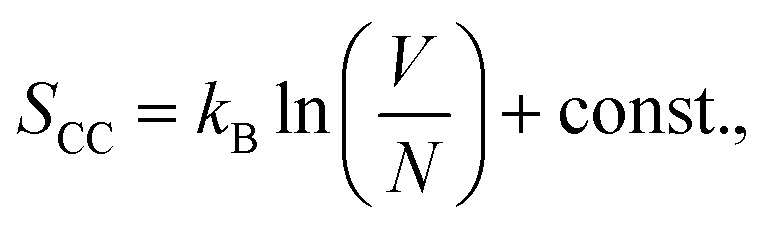
where *k*_B_ is the Boltzmann constant, *V* is the volume of the system, and *N* is the number of monomers. The real polymer chains may experience effects such as excluded volume, so we subtract the polymer volume *Nb* from the total volume as *b* is the effective volume (or area in 2D) of each monomer:
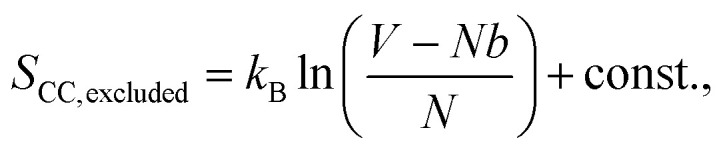
using the logarithmic property, we obtain
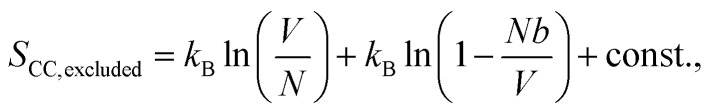
as *Nb*/*V* ≪ 1, we approximate ln(1 − *Nb*/*V*) ≈ −*Nb*/*V* and get
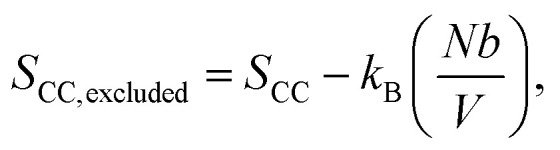
these effects will be applied to all types of films in the study.

When vanadium is introduced to the film, it behaves like an attractive potential source, pulling parts of the polymer chains towards the center. The vanadium creates a volume *V*_van_, with a radius determined by the strength of the electrostatic interaction between vanadium and the polymers. This shrinkage is opposed by effects like the excluded volume of the polymers and surface energy, leading to an equilibrium volume *V*_van1_. This occupied volume reduces the entropy, similar to how excluded volume in polymers decreases entropy. Moreover, the monomers bound to vanadium cannot contribute to the configurational state, further lowering the entropy. This is represented by *S*_bound_, which depends on the number of monomers in the chain that are constrained by the electrostatic source. Thus, the entropy in the soaked state becomes:
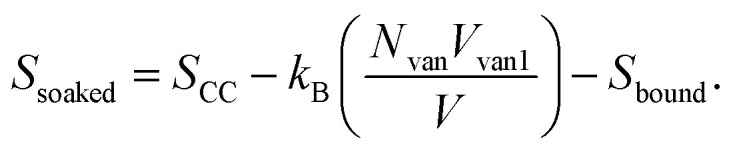


After irradiation, vanadium's behavior changes from attractive to repulsive; it pushes the polymer away from an effective volume *V*_van2_. This volume is controlled by electrostatic repulsion against the entropic forces that try to shrink the polymer. In this case, the number of monomers that contribute to the configurational states remains the same, but a portion of the space in the system is occupied by vanadium's effective volume *V*_van2_. Therefore, the entropy of the irradiated film takes the form:
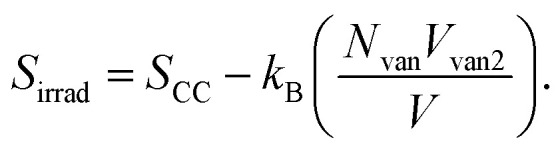


It follows that *S*_CC_ > *S*_irrad_ ≳ *S*_soaked_, which aligns with the RMS roughness values of the films as the higher entropy implies greater polymer entanglement, which maximizes the number of configurational states. Here, we can consider the roughness as a result of this local entanglement, which ultimately leads to the formation of entanglement peaks. However, it is important to note that the entropy for both the vanadium-soaked film and the irradiated film is lower than that of the control film. The exact order between them cannot be definitively concluded, but they are approximately similar – which also agrees with the RMS roughness value, with differences arising from *V*_van_, and the additional contribution *S*_bound_ for the vanadium-soaked case, which inversely varies with the number of monomers bound by the vanadium source. Since the polymer chains form the majority of the film, the entropy of the polymer chains reflects the degree of entanglement and is statistically represented by the RMS value of the entire film. This model is shown in the following subsections to also be consistent with the peak frequency and contact angle results of the polymers when the total partition function, which accounts for the effect of the vanadium atoms, is considered.

### 3.6. Peak frequency

As mentioned previously, the entropy of the CC films is the highest, followed by the irradiated film and the vanadium-soaked film. Note that the latter two are approximately in the same range. This is due to the fact that vanadium occupies some space, which in turn affects the polymer similarly to the excluded volume effect. However, as vanadium introduces electrostatic interactions, these interactions can alter thermodynamic quantities, changing the order between the vanadium-soaked case and the irradiated case.

The free energy, defined as *F* = −*k*_B_*T* ln *Z*_tot_, where *k*_B_ is the Boltzmann constant, *T* is the temperature, and *Z*_tot_ is the total partition function, follows the arrangement *F*_CC_ < *F*_soak_ < *F*_irrad_. Initially, *F*_polymer, CC_ < *F*_polymer, soak_ ≈ *F*_polymer, irrad_, but as the electrostatic interaction is introduced, with attractive forces in the vanadium-soaked case and repulsive forces in the irradiated case, terms like −|*E*_attract_| and +|*E*_repulsive_| arise. This leads to *F*_soak_ < *F*_irrad_.

Considering the total free energy, *F*_tot_, of the localized region where the polymer entangles and forms peaks, we can break it down into components as follows:^58^
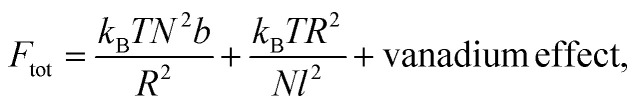
where the first term represents the excluded volume effect of the polymer, and the second term accounts for the random walk. Here, *N* is the number of monomers in the confined region, *b* is the excluded volume (or area in 2D), which can be approximated as *b* ≈ *l*^2^, as *l* is the characteristic size of the polymer, and *R* is the effective radius of this localized region. In this situation, we neglect the contribution from vanadium as the polymer effect dominates. We further simplify by considering only the leading order term *k*_B_*TN*^2^*b*/*R*^2^, as the condition *R* ≪ (*N*^3^*l*^2^*b*)^¼^ is satisfied, as shown in Table 1. Here, *R* is estimated to be half the distance between two peaks. We consider the farthest and nearest peaks in the three cases to cover all peak pairs. *N* is estimated from the mass density and the approximate volume confined to the peak. It was found that *R*/(*N*^3^*l*^2^*b*)^¼^ ∼ 0.001, which confirms *R* ≪ (*N*^3^*l*^2^*b*)^¼^ for all cases.

**Table 1:** Shows the *R*/(*N*^3^*l*^2^*b*)^¼^ values of the farthest and nearest peaks in each film type (CC, VCC, and VCC-IR films)

Cases	*R* (nm)	(*N*^3^*l*^2^*b*)^¼^	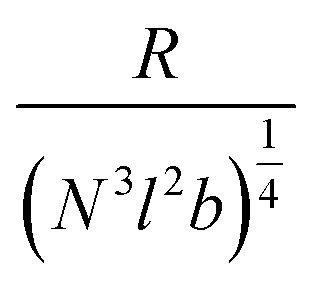
CC-nearest	355	765 780	0.00067
CC-farthest	1450	3 550 700	0.00041
VCC-nearest	335	157 590	0.00213
VCC-farthest	815	597 980	0.00136
VCC-IR-nearest	60	16 514	0.00363
VCC-IR-farthest	575	489 930	0.00117

**Fig. 7:**
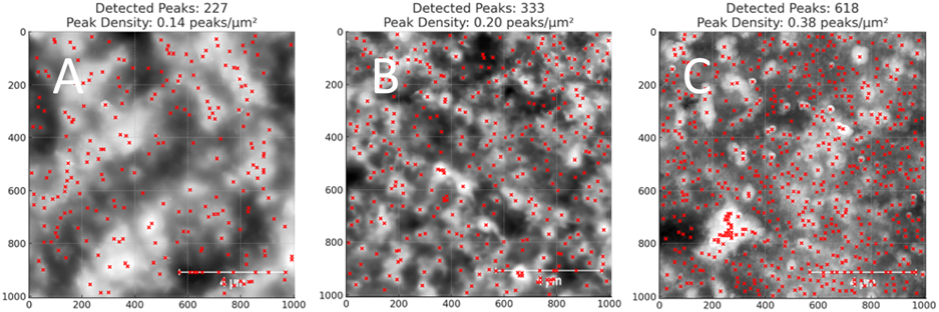
Show grayscale AFM images with detected peaks (red crosses) for (A) CC films, (B) VCC films, and (C) VCC-IR films. The original AFM images were processed in Python by converting them to grayscale, and peak detection was applied using scipy.signal.find_peaks to identify local maxima. The number of peaks and peak density (peaks per μm^2^) are shown for each image.

Thus, we proceed with:
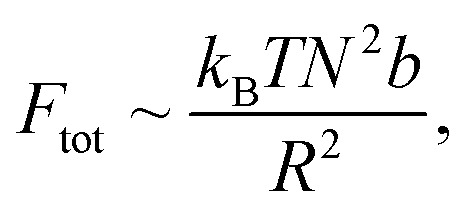
which implies:
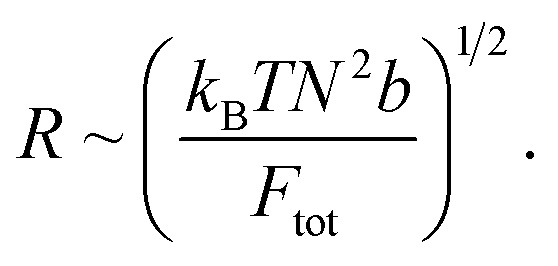


If we consider the curvature *κ* ≡ 1/*R*, we find:
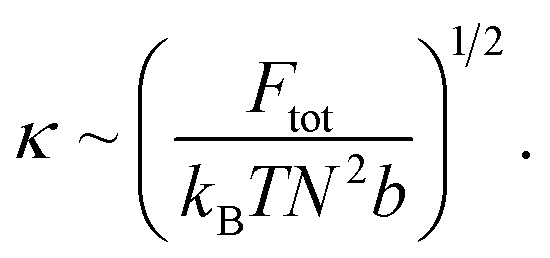


This suggests that *κ*_CC_ < *κ*_soak_ < *κ*_irrad_ where a lower *κ* indicates a more gradual valley between peaks, and a higher *κ* indicates a sharper valley. This is consistent with the peak frequency results, see Fig. 7, as the frequency *f* ∼ *N*_peak_/*L* ∼ *κ* and *N*_peak_ is the number of peaks along the length *L*.

### 3.7. Contact angle

**Fig. 8:**
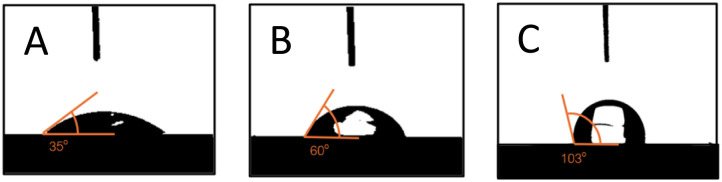
Show wettability properties with contact angles measured of (A) CC films, (B) VCC films, and (C) VCC-IR films.

For the contact angle, we argue that the free energy *F*, as defined and considered previously, is the energy available freely to do work and is utilized by the system for the work of adsorption, −*γ*_LV_(1 + cos *θ*).^59^ We obtain the contact angle using the following relation:
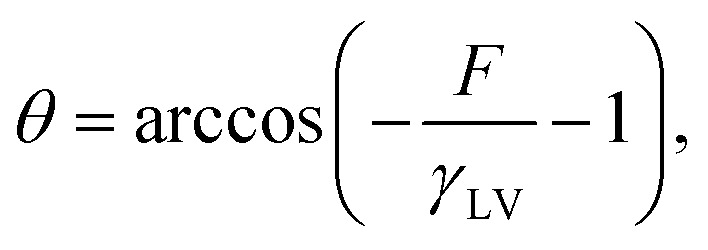
where *γ*_LV_ is the surface tension between the liquid and vapor phase. A higher free energy results in a lower value for the argument of the arccosine function, which in turn leads to a higher contact angle. Since *F*_CC_ < *F*_soak_ < *F*_irrad_, this implies that *θ*_CC_ < *θ*_soak_ < *θ*_irrad_ which agrees with the experimental results shown in Fig. 8A–C, where the contact angles follow the same trend.

### 3.8. Sample reversibility studies

**Fig. 9:**
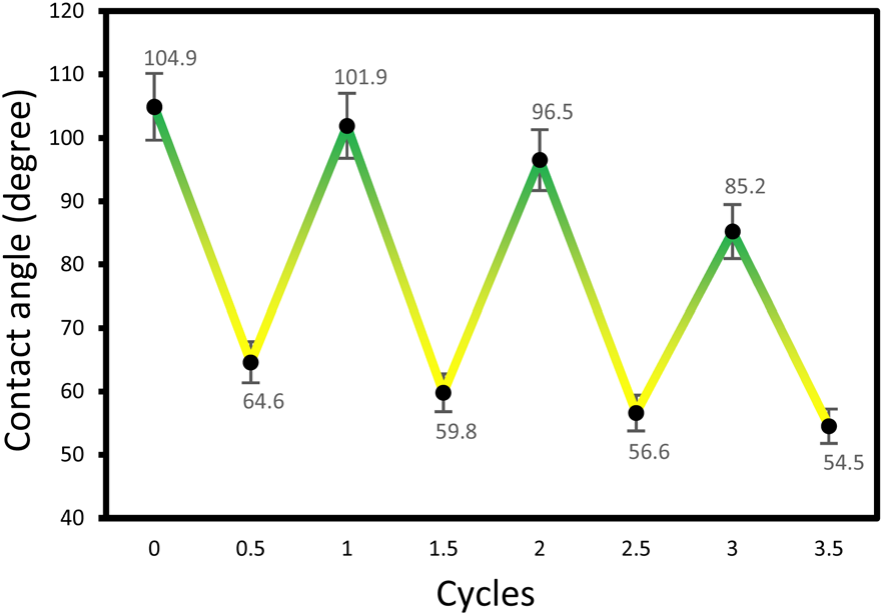
The on–off process illustrates the bifunctional nature of the material and its ability to switch between two states *via* photoreduction and thermal oxidation.

**Fig. 10:**
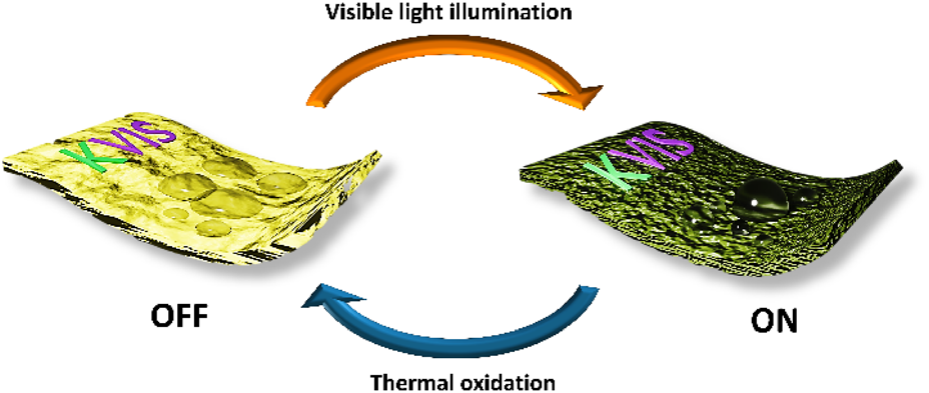
Reversibility measurements show an oscillatory behavior of VCC-IR films between hydrophobic and hydrophilic surface states when subjected to visible light illumination and thermal oxidation, respectively.

The reversibility of VCC-IR films was further examined through cyclical “on–off” experiments, alternating between thermal oxidation and visible light illumination (Fig. 9). A VCC-IR film sample underwent thermal oxidation at 60 °C for 8 hours, conditions chosen to induce surface oxidation and monitor the resulting structural changes. Following thermal oxidation, the contact angle of water was measured on the oxidized VCC-IR films. After contact angle measurement, the film was exposed to visible light for 8 hours, followed by another contact angle measurements. This process was repeated until a noticeable decline in sample performance was observed. The results of this experiment are summarized in Fig. 8. The contact angle was observed to oscillate between hydrophobic and hydrophilic states over 3.5 cycles before significant deactivation. Deactivation is likely caused by polymer degradation. The VCC-IR film's ability to alternate between a hydrophobic and a hydrophilic surface state using thermal oxidation and low-energy excitation represents a significant advancement for developing photoswitchable smart soft materials (Fig. 10).

## 4. Conclusions

In summary, vanadium-based metallopolymer films fabricated from starch and chitosan were extensively studied for their bifunctional surface properties. Soaking starch-chitosan films in metavanadate solution turns the film yellow. Subsequent white light illumination further shifts the film color to green. These color changes correspond to significant alterations in surface properties, as the sample became increasingly hydrophobic following vanadium incorporation into the biomaterial. A two-fold increase in the contact angle of water was observed between CC and VCC film samples (35° to 60°). XPS analysis shows a shift toward reduced vanadium species, diminishing electrostatic interactions between vanadium and biopolymer moieties. This frees up polymer stands, promoting polymer–polymer interaction and leading to surface restructuring as confirmed *via* AFM measurements. VCC-IR film samples were shown to switch from a hydrophobic state under visible light and revert to a hydrophilic state under thermal oxidation. This reversible bifunctionality of the film offers promising alternatives for smart-soft materials, with potential applications in membranes, material packaging, and food packaging.

## Data availability

The data supporting this article have been included as part of the ESI.†

## Author contributions

PS, CW, and NAA equally contributed to this study. These authors conducted all experiments in this study and were involved in providing most of the figures in the manuscript and ESI.† Additionally, they contributed to the manuscript write-up process. AT performed all SEM measurements, CK performed relevant AFM measurements, while SI performed XPS characterization of the samples. PN and NP fabricated samples during the revision process of this manuscript. PT proofread the manuscript, formed the theoretical model necessary in the interpretation of the structural mechanism, and provided figures central to the mechanism proposed. NC assisted with all experiments involved in this study and provided advice on the fabrication of the polymer samples. YM drafted the original manuscript, assisted with the experiments, and the spectroscopic assessment of the sample. Additionally, YM advised PS, CW, and NAA on all experiments conducted in this study.

## Conflicts of interest

The authors declare no conflict of interest.

## Supplementary Material

RA-015-D4RA08196J-s001
