# Supplementary material for: Bifunctional metavanadate promoted chitosan/cassava biopolymer films with photo-switchable wetting properties: unveiling the surface restructuring mechanism
Source: RSC Adv. 2025 Mar 11;15(10):7758–68. doi: 10.1039/d4ra08196j (PMC11895528; doi:10.1039/d4ra08196j)
Supplement: RA-015-D4RA08196J-s001 [file RA-015-D4RA08196J-s001.pdf]

Supplementary Information (SI) for RSC Advances.
This journal is © The Royal Society of Chemistry 2025

Supporting Information

Bifunctional Metavanadate Promoted Chitosan/Cassava Biopolymer Films with Photo-Switchable Wetting Properties: Unveiling the Surface Restructuring Mechanism

Pongpop Sangsawang,[‡a] Chayada Wisuttirattanamanee,[‡a] Nichaphat Auengaree,[‡a] Anawat Thivasasith,[b] Somlak Ittisanronnachai,[b] Chokchai Kaiyasuan,[c] Pawarisa Ngamroj[a], Natthakit Phophuttharaksa[a], Pattarapon Tanalikhit,[*d] Natputthiya Chavanalikigorn,[*a] and Yutichai Mueanngern[‡*a]

[a] Department of Chemistry, Kamnoetvidya Science Academy, 999 Moo 1, Pa Yup Nai, Wang Chan, Rayong, 21210, Thailand. E-mail: natputthiya.c@kvis.ac.th, yutichai.m@kvis.ac.th

[b] Frontier Research Center (FRC), Vidyasirimedhi Institute of Science and Technology 555 Moo 1, Pa Yup Nai, Wang Chan, Rayong 21210, Thailand

[c] School of Molecular Science and Engineering (MSE), Vidyasirimedhi Institute of Science and Technology 555 Moo 1, Pa Yup Nai, Wang Chan, Rayong 21210, Thailand

[d] Department of Physics, Korea Advanced Institute of Science and Technology, Daejeon 34141, Republic of Korea. E-mail: tanalikhit.p@kaist.ac.kr

‡ These authors contributed equally.

Table of contents

Fig. S1 Number of gas molecules adsorbed by 1 mm^2 of control (CC-films), Soaked (VCC-films), and Irradiated (VCC-IR-films) sample.

Fig. S2 Show (A) SEM-EDX quantification of biopolymer films soaked in metavanadate solutions from low to high concentration ranges. Sample C represents samples fabricated with conditions reported in the main manuscript. Corresponding contact angles are reported in (B).

Fig. S3 SEM-EDX analysis was collected normal to the sample surface after 3.5 photoreduction-thermal oxidation cycles.

Fig. S4 Show cross-sectional SEM micrographs of VCC-IR films after (A) no Thermal Oxidation (TO) and Photoreduction (PR), (B) 8 hours of TO, (C) 8 hours of TO and 8 hours of PR, (D) 16 hours of TO and 8 hours of PR.

Gas Adsorption

Gas chromatography measurements were carried out using a Shimadzu GC-14B gas chromatograph equipped with a capillary column and a flame ionization detector (FID). Controlled volumes of alcohol solutions consisting of methanol, ethanol, and isopropanol were mixed together in a 1:1:1 volume ratio in a single beaker and tightly sealed with parafilm. The beakers were left to sit for 30 minutes to allow for the alcohols to establish phase equilibrium in the head space above the respective liquids. Three separate beakers with the same alcohol concoction were prepared for CC, VCC, and VCC-IR-film samples. Each sample was suspended in the gas-phase head space of the liquid in its respective beakers. A luer lock gas-tight GC syringe was used to inject equal volumes of samples for analysis. The difference in peak areas, between the control beaker without a film sample and the beaker with a film sample, represents a semiquantitative assessment of the gas adsorption abilities for each film via the number of gas molecules.

The number of adsorbed gas molecules was divided by total area of the films' 2D surface area to calculate the number of gas molecules which can be adsorbed per 1 mm^2 of each film. The results are summarized in Fig. S1 below.

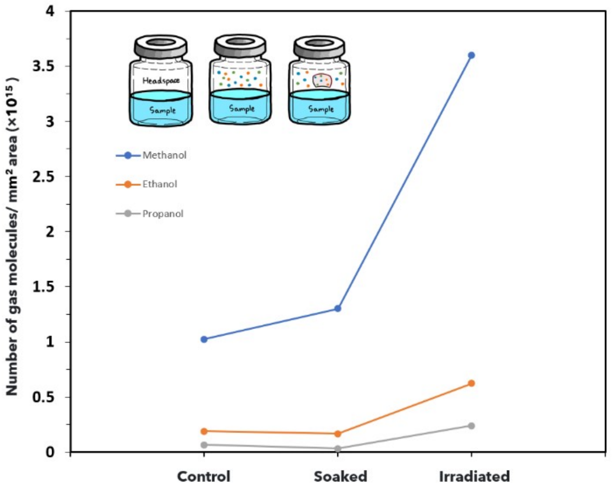

Fig. S1 Number of gas molecules adsorbed by 1 mm^2 of control (CC-films), Soaked (VCC-films), and Irradiated (VCC-IR-films) sample.

Vanadium Content

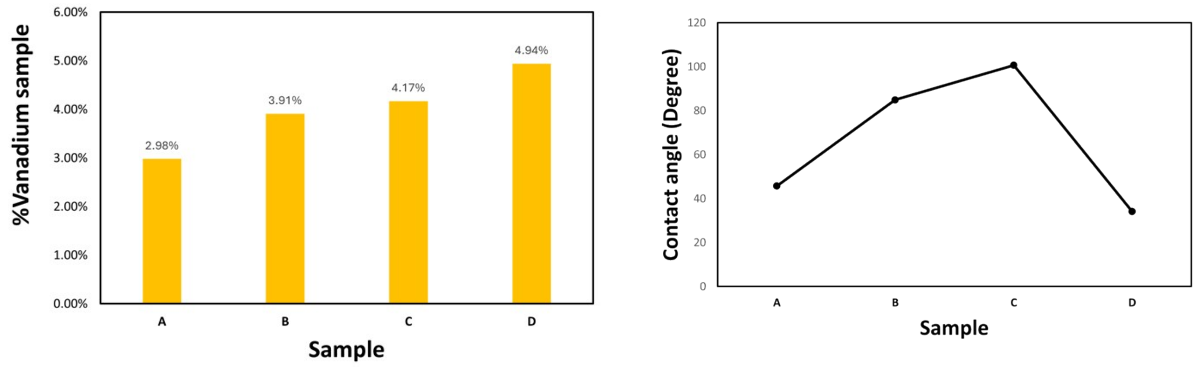

(A) **(B)**

Fig. S2 Show (A) SEM-EDX quantification of vanadium in biopolymer films soaked in metavanadate solutions from low to high concentration ranges. Sample C represents samples fabricated with conditions reported in the main manuscript. Corresponding contact angles are reported in **(B).**

To investigate our material property as a function of vanadium content, the hydrophobic properties of the biopolymer films were determined at various % vanadium content. All contact angles were measured after exposing the material to visible light irradiation, as previously reported in the main manuscript. The reported water contact angles of VCC-IR samples with vanadium content of 2.98%, 3.91%, 4.17%, and 4.93% were measured to as 45.7^{o}, 84.9^{o}, 100.6^{o}, and 34.1^{o}, respectively. At very low sodium metavanadate concentrations, approaching 0 M, the material immediately dissolves in the vanadium solution. As reported by Haddad et al. (reference 32, main manuscript), vanadium forms strong interaction with the biopolymer, yielding improved tensile strength and durability in water. However, in the absence of vanadium (or very low vanadium concentrations) the material behaves like pure chitosan-starch films. As the vanadium content increases, the film's hydrophobic properties is enhanced reaching a critical angle of 100.6^{o} at 4.17% vanadium. This can be explained by the increased interaction between vanadium and the film, which maximizes the free energy and results in the largest contact angle, as described by the following contact angle equation; this formula was previously discussed in Section 3.7 of the main manuscript:

$$\theta = \arccos\left(-\frac{F}{\gamma_{\mathrm{LV}}} - 1\right).$$

The sensitivity of the material's hydrophobic properties to small increases in vanadium content is interesting and is a subject for further studies. At higher vanadium content of 4.93% the material approaches smaller contact angle values approaching those of pure VO_x films (~40-50°) as have been reported by Khokhlova et al.[1]

Surface Morphology After Photoreduction-Thermal Oxidation Cycles

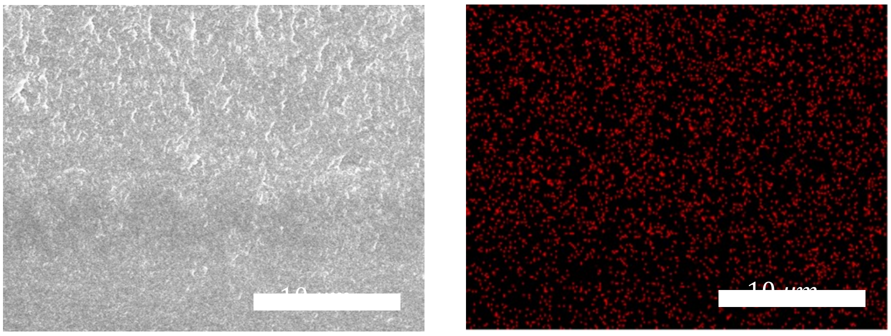

Fig. S3 SEM-EDX analysis was collected normal to the sample surface after 3.5 photoreduction-thermal oxidation cycles.

SEM/EDS analysis was conducted to examine the distribution of vanadium within the film after multiple thermal oxidations and photoreduction cycles. As shown in **Fig. S3**, the elemental mapping reveals that vanadium remains homogeneously distributed across the sample, even after repeated treatments. This indicates that vanadium remains within the polymer matrix throughout the process.

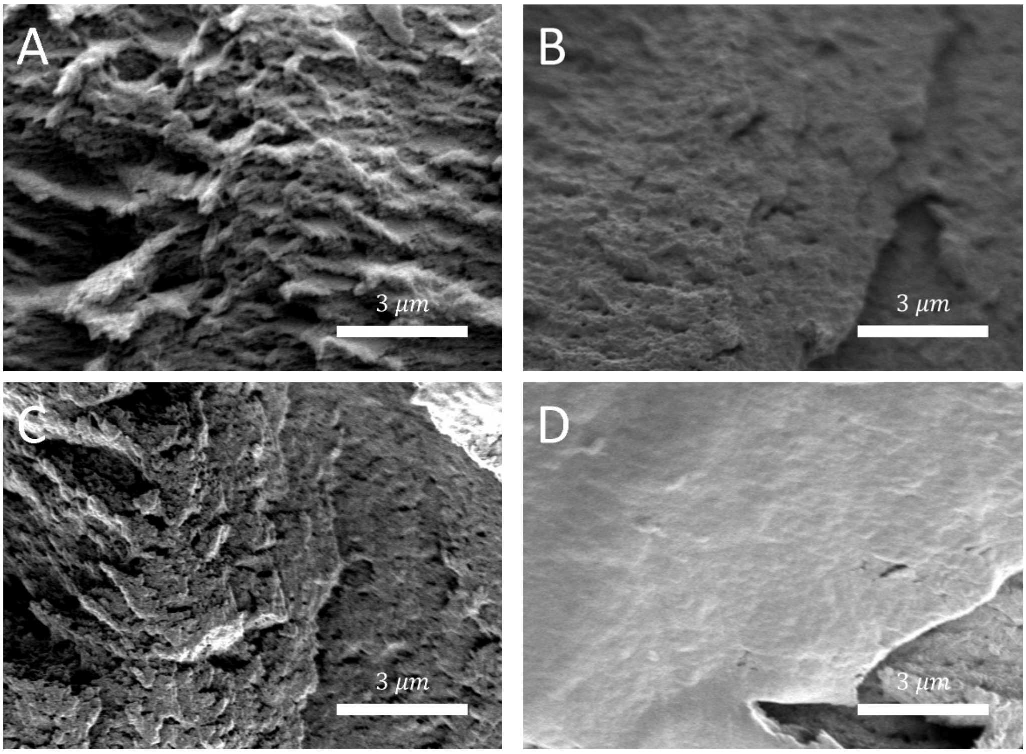

Fig. S4. Show cross-sectional SEM micrographs of VCC-IR films after (A) no Thermal Oxidation (TO) and Photoreduction (PR), (B) 8 hours of TO, (C) 8 hours of TO and 8 hours of PR, (D) 16 hours of TO and 8 hours of PR.

To further investigate the material's reversible physical properties, cross-sectional SEM analysis of film's surface morphology was performed for a select sample which underwent several thermal oxidation-photoreduction cycles. SEM images in (Fig. S4) reveal surface morphologies caused by thermal oxidation in-line with morphologies of VCC films reported in the main manuscript. Additionally, photoreduction leads to surface morphologies similar to those of VCC-IR film. These morphologies readily switch back and forth depending on the sample treatment utilized, indicating the reversibility of the surface morphology, which corroborates well with observed contact angle measurements in the main text.

Reference

1. Khokhlova, Maria, et al. "Controlling mesenchymal stem cell differentiation using vanadium oxide thin film surface wettability." *APL Materials* 11.7 (2023)
